# Enhancing beliefs and implementation of evidence-based practice among undergraduate nurses using a multi-component educational programme: a pre–post study

**DOI:** 10.1186/s12909-025-07121-x

**Published:** 2025-04-14

**Authors:** Filipa Pereira, Brigitte Lehmann-Wellig, Henk Verloo

**Affiliations:** 1 School of Health Sciences, HES-SO Valais/Wallis, Chemin de l’Agasse 5, Sion, CH-1950 Switzerland; 2 School of Health Sciences, HES-SO Valais/Wallis, Pflanzettastrasse 6, Viège, CH-3930 Switzerland; 3 School of Health Sciences, HES Valais/Wallis, Chemin de l’Agasse 5, Sion, CH-1951 Switzerland

**Keywords:** Evidence-based, Training, Nursing, Clinical practices, Regression analysis, Knowledge integration, Knowledge transfer

## Abstract

**Background:**

Using evidence-based practice (EBP) is one of the core skills that students should have acquired by the end of their Bachelor of Science in Nursing (BSN). However, little is known about how their beliefs about EBP and their frequency of implementation relate to multi-component educational programmes on this topic. This study aims to investigate the impact of a multi-component educational programme on nursing students’ beliefs about EBP and their frequency of implementation at the School of Health Sciences, HES-SO Valais.

**Methods:**

This quantitative, pre–post-design study compared undergraduate nursing students’ beliefs about EBP and their frequency of implementation before and after completing a multi-component educational programme on EBP. The programme included integrative workshops based on the steps of EBP held during their clinical internships throughout their three-year curriculum. The study occurred between September 2017 and June 2020: the start and end of their studies. The programme’s impact was measured using Melnyk et al.’s self-reported EBP Beliefs and Implementation Scales. Descriptive, comparative, correlational and regression statistics were computed to evaluate nurses’ responses and scores.

**Results:**

Ninety-five eligible first-year undergraduate nursing students were invited to participate in this EBP study and 81 completed the pre- and post-test questionnaires. Mean EBP scores improved significantly versus baseline on both the Beliefs (49.1 vs. 53.3; *p* < 0.001) and Implementation (1.7 vs. 9.0; *p* < 0.001) scales. Cronbach alphas for the EBP Beliefs scale were 0.799 pre-test (95% CI: 0.729, 0.858) and 0.869 post-test (95% CI: 0.823, 0.907). Cronbach’s alphas for the EBP Implementation scale were 0.804 pre-test (95% CI: 0.736, 0.861) and 0.939 post-test (95% CI: 0.918, 0.957). There were significant correlations between EBP Beliefs and Implementation scores (*p* < 0.001). Linear regression analysis showed that the programme’s theory-based component contributed significantly more than clinical internships to raising EBP Beliefs and Implementation scores.

**Conclusions:**

The multi-component educational programme on EBP improved undergraduate nursing students’ EBP Beliefs and Implementation scores. Future research should investigate means of optimally integrating EBP into undergraduate nursing curricula and explore nursing students’ intentions to implement EBP in clinical practice.

**Supplementary Information:**

The online version contains supplementary material available at 10.1186/s12909-025-07121-x.

## Background

Evidence-based practice (EBP) can no longer be considered optional for healthcare professionals [1, 2]. Their clinical decision-making in every domain of practice should always be based on the best available knowledge and best practices [1, 3]. Healthcare professionals should, therefore, acquire some theoretical and empirical knowledge about the concept of EBP, as well as the skills necessary to implement it [1]. EBP combines the individual’s unique clinical expertise with the best levels of evidence resulting from rigorous, well-designed research studies [4]. In addition to medicine, several other disciplines are interested in the EBP movement, including nursing, physiotherapy, occupational therapy, social work and even education [4]. The use of the same principles and concepts across various healthcare domains gave birth to the generic term of evidence-based practice [5]. Sackett et al.. defined EBP as a complex process that considers the existence of several simultaneous sources of information—scientific evidence, clinical expertise and patient preferences—to guide clinical decision-making for the provision of the best possible patient care (including the safety of care) and to measure that care’s effects [6]. Fineout-Overholt, Melnyk [7] and Berryman [8] added that EBP was a problem-solving approach for achieving best practices that consciously used the best available evidence when making decisions about patient care or therapies. Several advantages resulting from using EBP in decision-making have been documented, for patients, healthcare professionals and healthcare systems [1, 9]. For patients, the adequate use of evidence has been shown to contribute to more appropriate clinical decisions and to less exposure to ineffective or even dangerous interventions [10, 11]. For nurses making clinical decisions, the continual developments in scientific thinking and research skills have improved their ability to more systematically judge the benefits and effectiveness of the care given [12]. Awareness of the importance of EBP should imbue healthcare professionals with the scientific spirit essential to making the implementation of best practices a routine part of their daily clinical work [13, 14, 15]. This implies asking oneself the right questions, searching for evidence, examining all the possibilities and critically evaluating one’s own ideas [16]. Using EBP encourages the rational analysis of ideas, principles, and conclusions and the resolution of everyday clinical problems [17, 18]. The skills that healthcare professionals need in order to integrate EBP into their daily practice must be built up from the very beginning of their training [19]. Preparing student nurses for clinical practice and ensuring that they have a minimum set of core skills on graduation is assured by their undergraduate education curriculum [20]. It is essential to educate undergraduate nursing students on EBP to improve their knowledge about it, to strengthen their beliefs regarding its benefits to patients and nurses, and to enhance their self-efficacy in implementing it [21]. To reach these outputs, educational processes must be improved and should focus more on the beliefs and implementation of EBP. There is consistent evidence showing that although undergraduate nursing students have positive beliefs about EBP and its value in patient care, they also report on the many challenges of actually implementing it in clinical practice [22]. One mixed-methods study of 118 undergraduate nursing students in America revealed that they found it difficult to distinguish between EBP and research. They were able to search for evidence but were less able to integrate new evidence to plan changes to EBP or disseminate best practices [23]. An observational study conducted among undergraduate nursing students in Saudi Arabia reported positive beliefs about EBP but a low mean score on the EBP-Implementation scale (just 22/72). Several factors have been reported to influence the implementation of EBP, such as age, sex, awareness of EBP and training on EBP [24]. A recent survey of a large international sample of undergraduate nursing students from India, Saudi Arabia, Nigeria and Oman reported that the lack of readily available recent publications containing evidence and the lack of time to implement that evidence in the appropriate clinical speciality were important barriers to implementing EBP [25].

The University of Applied Sciences and Arts Western Switzerland (HES-SO) recently integrated an educational programme on EBP into the curriculum of its Bachelor of Sciences in Nursing (BSN) to improve students’ knowledge and implementation competencies in this domain. In parallel to their classroom studies, during their practical training and internships, students repeatedly encounter problematic clinical situations from which emerge more clinical and professional questions [26, 27]. Melnyk et al.. (2009) and Kim et al.. (2019) showed that training programmes on EBP developed student nurses’ sense of professional responsibility and increased their level of satisfaction during their practical training and throughout their studies [28, 29]. According to the latest reforms instituted across Switzerland’s universities and schools of health sciences, during their training, nursing students are supposed to use scientific research and reflective practices, critically search for, analyse and evaluate scientific literature, and then share their knowledge with their peers. However, literature exists that documents nursing students’ negative attitudes and beliefs about research and how they question the utility of EBP for clinical decision-making [30, 31]. Indeed, some contemporary authors have decried a lack of consistency between the training about EBP given in schools of health sciences and its use in practical training [32, 33]. Even though nursing students regularly encounter the concept of EBP during their academic studies, the impact of those encounters has never been well explored or fully documented [34]. With the goal of evaluating the evolution in undergraduate nursing students’ beliefs about EBP and their frequency of implementation,, we conducted a pre–post study among them using the EBP Beliefs (EBP-B) and EBP Implementation (EBP-I) scales developed by Melnyk et al. [35]. We also explored whether the theory-based component of their undergraduate curriculum contributed more to raising EBP-B and EBP-I scores than their practical internships in acute or long-term care. Finally, we investigated how well the EBP-B scores predicted EBP-I scores at the start and end of the study.

### Framework for the educational intervention on EBP

**Fig. 1:**
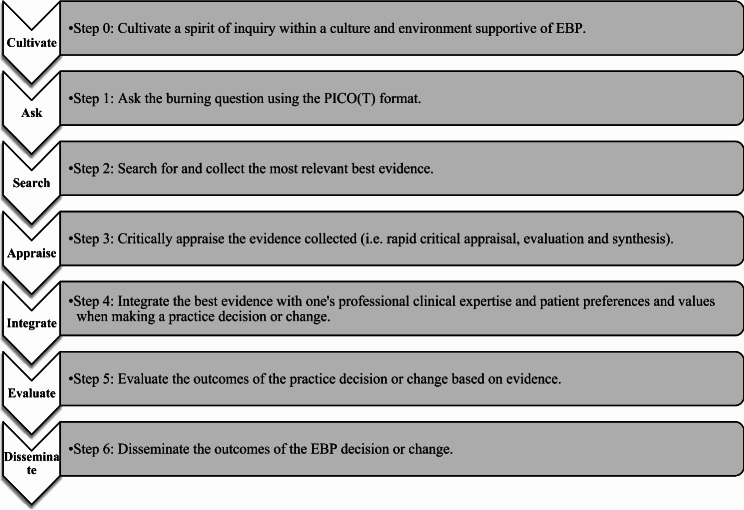
The steps in the EBP process according to Melnyk and Fineout-Overholt [1]

The present study referred to Melnyk and Fineout-Overholt’s seven-step process (2019) to guide healthcare professionals trying to integrate the most current and relevant research into their clinical practice [1]. The seven steps are presented in Fig. 1 and were used as a framework to design our multi-component educational programme.

Based on Melnyk and Fineout-Overholt, this study defined beliefs about the value of EBP as the student’s attitude towards the importance and utility of EBP in their daily practice. EBP implementation refers to the process of integrating the best available research evidence with personal professional clinical expertise and patient preferences into clinical practice to improve patient outcomes [1].

## Methods

### Study design

A quantitative pre–post study was conducted among undergraduate BSN students to explore changes in their beliefs about EBP and their frequency of implementation. These changes were measured using their self-reported EBP-B and EBP-I scale scores, as developed by Melnyk et al. [35]., before and after a multi-component educational programme on EBP. This study was conducted and reported according to the recommendations of *Transparent Reporting of Evaluations with Non-randomized Designs (TREND) statement* [36].

### Participants and sampling

In September 2017, all the first-year BSN students at the University of Applied Sciences and Arts Western Switzerland’s School of Health Sciences in the canton of Valais were invited to participate in the study. This was regardless of their previous educational or professional trajectory. Students were enrolled at both training sites (one French-speaking and one German-speaking). The exclusion criteria were being a postgraduate or an Erasmus student. This was a non-probability convenience sample.

### A multi-component educational programme on EBP

During their three-year BSN curriculum, all the nursing students participated in a multi-component educational programme on EBP comprising three educational approaches (Table 1).

**Table 1:** Description of the multi-component educational programme on EBP

Multi-component educational programme on EBP	
Practice-based component	Six clinical internships (60 ECTS)
Theory-based component	Standard curriculum on research methodology and EBP (20 ECTS)
	Six EBP workshops during the clinical internships (36 h)

### Clinical internships

The BSN’s three-year curriculum includes six clinical internships, each worth 10 European Credit Transfer and Accumulation System (ECTS) credits: four internships of six weeks and two of eight weeks, at 40 h per week. Each undergraduate student does a variety of clinical internships representing different fields of nursing: acute care, community healthcare and long-term care (inpatient, outpatient and at home) for patients of all ages. Clinical internships were complemented by ‘back to school’ activities lasting three hours in week three or four of each internship. These activities occurred at the School of Health Sciences and included simulations, practical workshops and virtual reality sequences that enhance links between theory and practice. Students brought back complex pathophysiological and nursing care situations that they experienced during their internships, and these were explored with one of the school’s experts in the field based on best evidence.

The types of clinical internships were assessed using Hutchinson et al..’s classification for healthcare settings: (i) acute care (hospital care), (ii) long-term care (rehabilitation, nursing homes, long-term healthcare facilities) and (iii) community healthcare (outpatient primary care and community healthcare settings) [37].

### The standard BSN curriculum on research methodology and EBP

The standard BSN curriculum comprises 500 h divided into in-class or mentored training and personal work) focusing on EBP and research methodology. Courses take place during the second and third year of the BSN and aim to develop students’ critical thinking and analytical skills by teaching them how to assess and interpret research findings to make informed decisions in clinical settings. This standard programme also aims to equip students with the research skills to ensure that future nurses can apply the latest evidence to patient care, keeping practice up to date with the latest guidelines and studies and thus improving patient outcomes.

### EBP workshops

The EBP workshops are an innovative approach to teaching the EBP process [1]. Six workshops occurred during the student nurses’ clinical internships, integrating the alternating theoretical components of their education and the clinical reality they experienced during those internships. The workshops were led by EBP experts and, in total, comprised 18 h of in-class workshops and 18 h of student homework (Table 2). Sessions were conducted with groups of 8–10 students, and real-life clinical cases experienced by the students during their clinical internships were used to apply the steps of EBP. In addition to the EBP steps, the workshops covered topics related to EBP, such as a Student Journal Club [38] and the use of evidence-based handovers or handoffs [39]. Given that steps 5 and 6 (Fig. 1) could not be performed in a workshop without a real implementation environment, they have been replaced by other EBP-related activities: a journal club and best practices in nursing handovers [1].

The structure of the entire curriculum of students involved in the study can be found in Supplementary File 1. The minimum attendance requirement of the students participating in the study was 80%.

**Table 2:** EBP workshops and their content during the BSN curriculum

Period	Content	Length	Learning Objectives
Bachelor 1st year 2017/2018	Workshop 1: - Step 0: Reflective Practice and ‘Critical Thinking’ - Step 1: Development of a PICOT/PEOT research question	3 h homework and 3 h classwork during week 3 of 1st clinical internship	- Define reflective practice and its role in clinical reasoning - Develop critical thinking skills for nursing practice by analysing clinical situations - Formulate a structured and relevant PICOT/PEOT research question
	Workshop 2: - Steps 0 to 1 - Step 2: Search for and collect the most relevant best evidence	3 h homework and 3 h classwork during week 3 of 2nd clinical internship	- Apply effective search strategies using scientific databases - Identify and select high-quality, relevant evidence - Develop autonomy in retrieving evidence to answer a clinical question
Bachelor 2nd year 2018/2019	Workshop 3: - Steps 0 to 2 - Step 3: Critical appraise the selected studies	3 h homework and 3 h classwork during week 3 of 3rd clinical internship	- Apply critical appraisal tools to evaluate research quality. - Distinguish between different levels of evidence. - Assess the validity, reliability, and applicability of selected studies.
	Workshop 4: - Step 0 to 3 - Step 4: Applying/integrating the evidence into practice	3 h homework and 3 h classwork during week 3 of 4th clinical internship	- Synthesize research findings to inform clinical decision-making - Integrate patient preferences and clinical expertise with research evidence
Bachelor 3rd year 2019/2020	Workshop 5: Journal Club by nursing students	3 h homework and 3 h classwork during week 4 of 5th clinical internship	- Critically discuss research articles in a peer-led setting - Develop skills in presenting and debating research findings - Enhance confidence in applying EBP through collaborative learning
	Workshop 6: Evidence-Based Nursing Handover	3 h homework and 3 h classwork during week 4 of 6th clinical internship	- Communicate research-based clinical decisions effectively - Structure nursing handovers using an EBP approach - Promote a culture of EBP in clinical teams

Note. PICOT = Population, Intervention, Comparator, Outcomes, Time; PEOT = Population, Event, Outcomes, Time

### Research instruments

The present study selected the EBP Beliefs (EBP-B) and EBP Implementation (EBP-I) scales, originally developed by Melnyk et al. [35]., because they: (i) exist in validated, culturally adapted French- and German-language versions [40, 41] (which could be used at our two research sites); (ii) have recognised excellent psychometric properties; (iii) are easy to use within the framework of a large-scale study; and (iv) other studies have successfully used the EBP-B and EBP-I scales to explore EBP and its use by nursing students [2, 23]. Although Melnyk et al. have developed specific versions of the EBP-B and EBP-I scales for students [1], these have not yet been translated and validated in French and German, the languages used in the present study. For this reason, we opted to use the versions designed for nurses, as they remain conceptually close to those intended for students.

#### Instruments

The EBP-B is a 16-item questionnaire using a 5-point Likert scale to measure individuals’ beliefs about the value of EBP and their ability to implement it [35]. Possible responses ranged from ‘1 = Strongly disagree’ to ‘5 = Strongly agree’. Item scores were summed, so total scores could range from 16 to 80, with higher scores indicating stronger beliefs in the value of EBP. The EBP-I is a 17-item questionnaire using a 5-point frequency scale to measure how often students had performed specific EBP processes in the previous eight weeks or during their last clinical internship [35]. Possible responses ranged from ‘0 = No activity during the last 8 weeks’ to ‘4 = Activity done 8 times or more’. Total scores could range from 0–68, with higher scores indicating the more frequent use of EBP processes. Higher total scores reflected the more frequent use of the steps or components of EBP [35]. The EBP-B and EBP-I scales have demonstrated good psychometric properties in previous studies, particularly regarding internal consistency: Cronbach alphas were α = 0.90 and α = 0.96, respectively [35, 40, 42]. Both the French and German translations were tested for reliability and validity by the present study’s authors [40, 41, 43]. The French version was translated in English and is available in Supplementary File 2.

#### Sociodemographic data

Sociodemographic and professional data were collected on age, sex, types of clinical internship (acute care, long-term care facilities, community care), and previous exposure to EBP and in which circumstances.

### Data collection procedure

The study was approved by the School of Health Sciences of HES-SO Valais / Wallis’s Internal Ethics Board review committee (IB-UAS-Nur&Phy/001/17). Data were collected close to the start of the first-year undergraduate nursing students’ curriculum (September 2017). The research team chose a moment when a maximum number of students would be in attendance. The students received no prior announcement about the information session in order to avoid any methodological bias in the data collection. At the information session, the research team gave the students a brief introduction to the study, including an explanation of its goals, methods and ethical considerations. Those who consented to enrol and participate in the study completed our paper questionnaires to assess their beliefs about EBP and their frequency of implementation at baseline. These were measured again at the end of their three-year education programme (June 2020). The EBP-B and EBP-I scales were completed in French or German, at our sites in the canton of Valais’ French- and German-speaking regions, respectively. The investigators insisted on the importance of completing the questionnaire fully in order to avoid missing data.

### Data analyses

A database was built using Excel^®^ spreadsheet software (2018) and completed with the collected data on sociodemographic variables and the item and total scores from the EBP-B and EBP-I questionnaires at baseline and after the EBP educational programme. A missing value analysis was conducted to spot any patterns of missing data. Questionnaires with missing sociodemographic data or with responses to fewer than 80% of the EBP scales’ items were excluded. All the data were analysed regarding their parametric characteristics (normal distribution, homogeneity, linearity) before moving on to complementary analyses. Parametric statistical tests were applied to normally distributed variables, and non-parametric statistical tests were chosen for variables with non-normal distributions. We described the sample and calculated the means (standard deviations) of the item and total scores for the EBP-B and EBP-I scales. We also made inferential analyses using the statistical tests appropriate for the different types of variables. Because all the items on the EBP-B and EBP-I scales showed normal distributions, based on a Shapiro–Wilk analysis [44], they were analysed using a two-tailed paired t-test. If there were any statistically significant associations between the final EBP-I scores and the EBP-B scores at the start and end of the study, then Pearson correlations were computed to examine their intercorrelations. The floor and ceiling effects of the responses were analysed using a cut-off of a 20% positive or negative skewness distribution in relation to each item’s mean [45, 46]. The questionnaires’ internal consistency was computed using Cronbach’s alpha, which has a normal range between 0.00 and + 1.00, with higher values reflecting better internal consistency [47]. Multivariate linear regressions were calculated to find the best predictors of total EBP-B and EBP-I scores at the end of the study in relation to clinical internships in acute care and long-term care and to the standard programme on research methodology and EBP. We did not include clinical internships in community care because the sample of students was so small. We also investigated how well total EBP-B scores at baseline predicted total EPB-I scores at baseline and at the end of the study. Data were analysed using the Statistical Package for Social Sciences (IBM SPSS statistics version 29.0, IBM Ltd, Portsmouth, UK). The level of significance was set at *p* ≤ 0.05, two-tailed, with a 95% confidence interval (CI).

## Results

### Participation

**Fig. 2:**
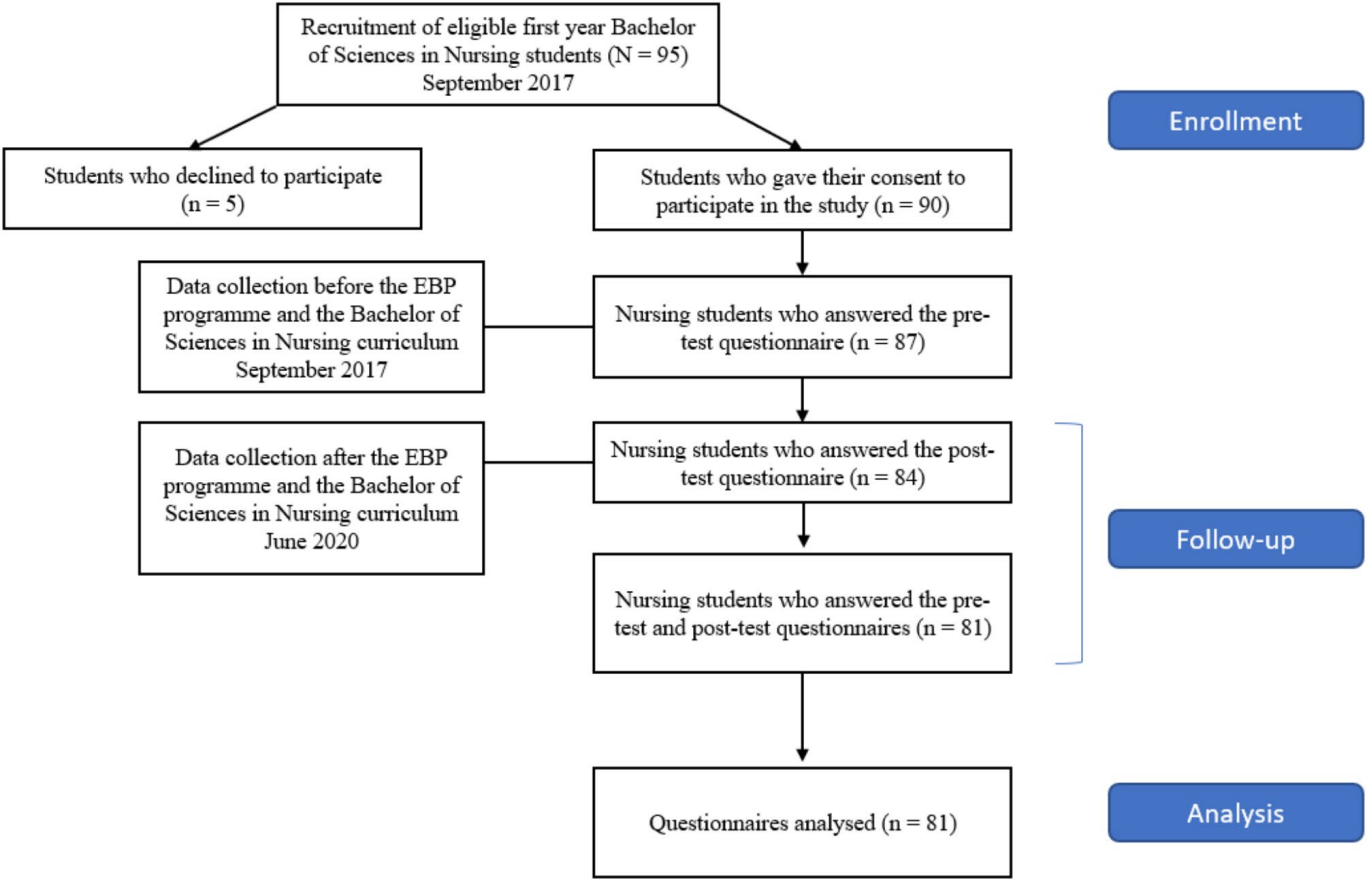
Sample participation and questionnaire completion flowchart

Of 95 first-year students invited to participate in the study, 90 (94.7%) accepted and 5 declined to participate because they were “not interested in the study”. The baseline questionnaires were completed by 87 (91.6%) students before their multi-component educational programme on EBP. Three students left the BSN curriculum in the first or second year of BSN, and 84 completed the questionnaires at the end of the multi-component educational programme on EBP. In the end, 81 questionnaires were more than 80% completed and thus useable for our analysis. Figure 2 presents the study participation and data collection flowchart over the course of the BSN curriculum.

### ***Participants’ characteristics***

The sociodemographic data only included those participants whose questionnaires were more than 80% completed. At baseline, most participants were women (82.7%), mean age was 21.7 (SD = 3.9) years old, and 74.1% reported never having been exposed to the concept of EBP before, though 25.9% had previously heard about EBP during their pre-bachelor training. Frequencies and percentages are presented in Table 3.

Participants’ clinical internships were split into acute, long-term, or community healthcare setting categories based on Hutchinson et al..’s classification system [45]. Categorisation was determined by evaluating in which setting students had completed more than half of their six clinical internships. In the case of an equal distribution of internship settings, the acute healthcare setting was arbitrarily selected.

**Table 3:** Participants sociodemographic characteristics and internship settings (*n* = 81)

Sociodemographic characteristics and internship settings	Frequencies
Sex	
Men (%)	14 (17.3)
Women (%)	67 (82.7)
Age ^1^	
Average (SD)	21.7 (3.9)
Median (IQR 1–3)	20 (19–23)
Min–Max	18–42
Previous exposure to EBP ^1^	
No	60 (74.1)
Yes	21 (25.9)
Clinical internships ^2^	
Most internships (≥ 4) in acute healthcare settings	41 (50.6)
Most internships (≥ 4) in long-term healthcare settings	32 (39.5)
Most internships (≥ 4) in community healthcare settings	8 (9.9)

^1^ Collected at the beginning of the study (September 2017)

^2^ Collected at the end of the study (June 2020)

### Mean EBP-B and EBP-I item scores

With the exception of the mean scores for “I believe that EBP is difficult”, which did not show a significant difference (*p* = 0.320), all of the EBP-B and EBP-I scale items showed significantly higher mean post-test scores than mean pre-test scores (*p* < 0.05). Tables 4 and 5 present the two-tailed, paired sample t-test results for the differences between the EBP-B and EBP-I scales’ pre-test and post-test mean scores. No floor or ceiling effects were found in the EBP-B and EBP-I scales based on a skewness distribution greater than 20% compared to the mean.

**Table 4:** Mean scores for each EBP-B scale item and t-test results for paired samples (*n* = 81)

EBP-B items		Pre-test mean (SD)	Post-test mean (SD)	t**	95% CI	*p*- value
1	I believe that EBP results in the best clinical care for patients	3.67 (0.71)	4.04 (0.66)	-4.450	-0.536, -0.205	< 0.001
2	I am clear about the steps of EBP	2.21 (0.96)	2.96 (1.16)	-5.603	-1.021, -0.486	< 0.001
3	I am sure that I can implement EBP	2.77 (1.05)	3.27 (0.96)	-4.862	-0.713, -0.299	< 0.001
4	I believe that critically appraising evidence is an important step in the EBP process	3.69 (0.75)	3.91 (0.76)	-2.530	-0.397, -0.047	0.013
5	I am sure that evidence-based guidelines can improve clinical care	3.77 (0.71)	4.07 (0.77)	-3.342	-0.492, -0.125	0.001
6	I believe that I can search for the best evidence to answer clinical questions in an efficient way	3.15 (0.82)	3.43 (0.80)	-3.042	-0.470, -0.098	0.003
7	I believe that I can overcome barriers to implementing EBP	3.14 (0.82)	3.33 (0.71)	-2.375	-0.363, -0.032	0.020
8	I am sure that I can implement EBP in a time-efficient way	2.93 (0.97)	3.16 (0.93)	-2.315	-0.436, -0.033	0.023
9	I am sure that implementing EBP will improve the care that I deliver to my patients	3.64 (0.73)	3.95 (0.76)	-3.964	-0.464, -0.154	< 0.001
10	I am sure about how to measure the outcomes of clinical care	2.57 (0.91)	2.98 (0.97)	-3.990	-0.611, -0.204	< 0.001
11	I believe that EBP takes too much time (inversed scoring)	3.16 (0.71)	2.93 (0.74)	3.665	0.107, 0.362	< 0.001
12	I am sure that I can access the resources needed to implement EBP	3.14 (0.72)	3.35 (0.78)	-3.114	-0.344, -0.076	0.003
13	I believe that EBP is difficult (inversed scoring)	2.77 (0.79)	2.72 (0.84)	1.000	-0.049, 0.148	0.320
14	I know enough about implementing EBP to make practice changes	2.59 (0.92)	2.80 (0.89)	-2.625	-0.369, -0.051	0.010
15	I am confident about my ability to implement EBP where I work	2.73 (0.95)	2.94 (0.94)	-2.922	-0.353, -0.067	0.005
16	I believe the care that I deliver is evidence-based	3.20 (0.90)	3.47 (0.85)	-3.643	-0.420, -0.123	< 0.001

Note. SD = standard deviation; * Significant difference *p* ≤ 0.05; ^**^t-test for paired samples

**Table 5:** Mean scores for each EBP-I scale item and t-test results for paired samples (*n* = 81)

EBP-I items		Pre-test mean (SD)	Post-test mean (SD)	t^**^	95% CI	*p*-value
1	Collected data on a patient problem	0.05 (0.35)	0.65 (1.04)	-5.610	-0.820, -0.390	< 0.001
2	Used evidence to change my clinical practice	0.09 (0.28)	0.70 (1.01)	-5.658	-0.834, -0.400	< 0.001
3	Informally discussed evidence from a research study with a colleague	0.01 (0.11)	0.57 (0.89)	-5.590	-0.753, -0.358	< 0.001
4	Read and critically appraised a clinical research study	0.23 (0.58)	0.53 (0.87)	-3.344	-0.473, -0.120	< 0.001
5	Shared an EBP guideline with a colleague	0.47 (0.90)	1.42 (1.48)	-5.584	-1.289, -0.612	< 0.001
6	Critically appraised evidence from a research study	0.06 (0.37)	0.27 (0.52)	-3.114	-0.344, -0.076	0.003
7	Changed practice based on patient outcome data	0.11 (0.47)	0.44 (0.77)	-3.408	-0.528, -0.139	< 0.001
8	Promoted the use of EBP to my colleagues	0.06 (0.37)	0.46 (0.84)	-3.819	-0.601, -0.189	< 0.001
9	Shared evidence from a research study with a multidisciplinary team member	0.10 (0.44)	0.31 (0.64)	-2.690	-0.365, -0.055	0.009
10	Accessed the Cochrane database of systematic reviews	0.05 (0.22)	0.31 (0.66)	-3.406	-0.411, -0.108	0.001
11	Shared the outcome data collected with colleagues	0.21 (0.52)	0.60 (0.89)	-4.060	-0.589, -0.201	< 0.001
12	Evaluated the outcomes of a practice change	0.01 (0.11)	0.67 (1.21)	-4.884	-0.921, -0.388	< 0.001
13	Used an EBP guideline/systematic review to change clinical practice where I work	0.04 (0.33)	0.51 (0.92)	-4.789	-0.664, -0.274	< 0.001
14	Shared evidence from a study in the form of a report/presentation to > 2 colleagues	0.10 (0.37)	0.35 (0.64)	-3.970	-0.371, -0.123	< 0.001
15	Shared evidence from a research study with a patient/family member	0.09 (0.39)	0.44 (0.93)	-3.702	-0.551, -0.166	< 0.001
16	Generated a PICOT question about my clinical practice	0.04 (0.18)	0.38 (0.72)	-4.277	-0.507, -0.185	< 0.001
17	Evaluated a care initiative by collecting patient outcome data	0.06 (0.24)	0.37 (0.73)	-4.442	-0.477, -0.170	< 0.001

Note. SD = standard deviation; * Significant difference *p* ≤ 0.05; ** *t*-test for paired samples

To assess whether the data from the 16-item and 17-item pre-test and post-test EBP-B and EBP-I scales were reliable, we investigated their internal consistency using Cronbach’s alpha. The Cronbach alpha for the pre-test EBP-B scale was 0.799 (95% CI: 0.729, 0.858), and for the post-test EBP-B scale it was 0.869 (95% CI: 0.823, 0.907), so both displayed good internal consistency [39]. The Cronbach alpha for the pre-test EBP-I scale was 0.804 (95% CI: 0.736, 0.861), and for the post-test EBP-I scale it was 0.939 (95% CI: 0.918, 0.957), so both displayed good internal consistency [35].

### Total pre- and post-test EBP-B scale scores

**Fig. 3:**
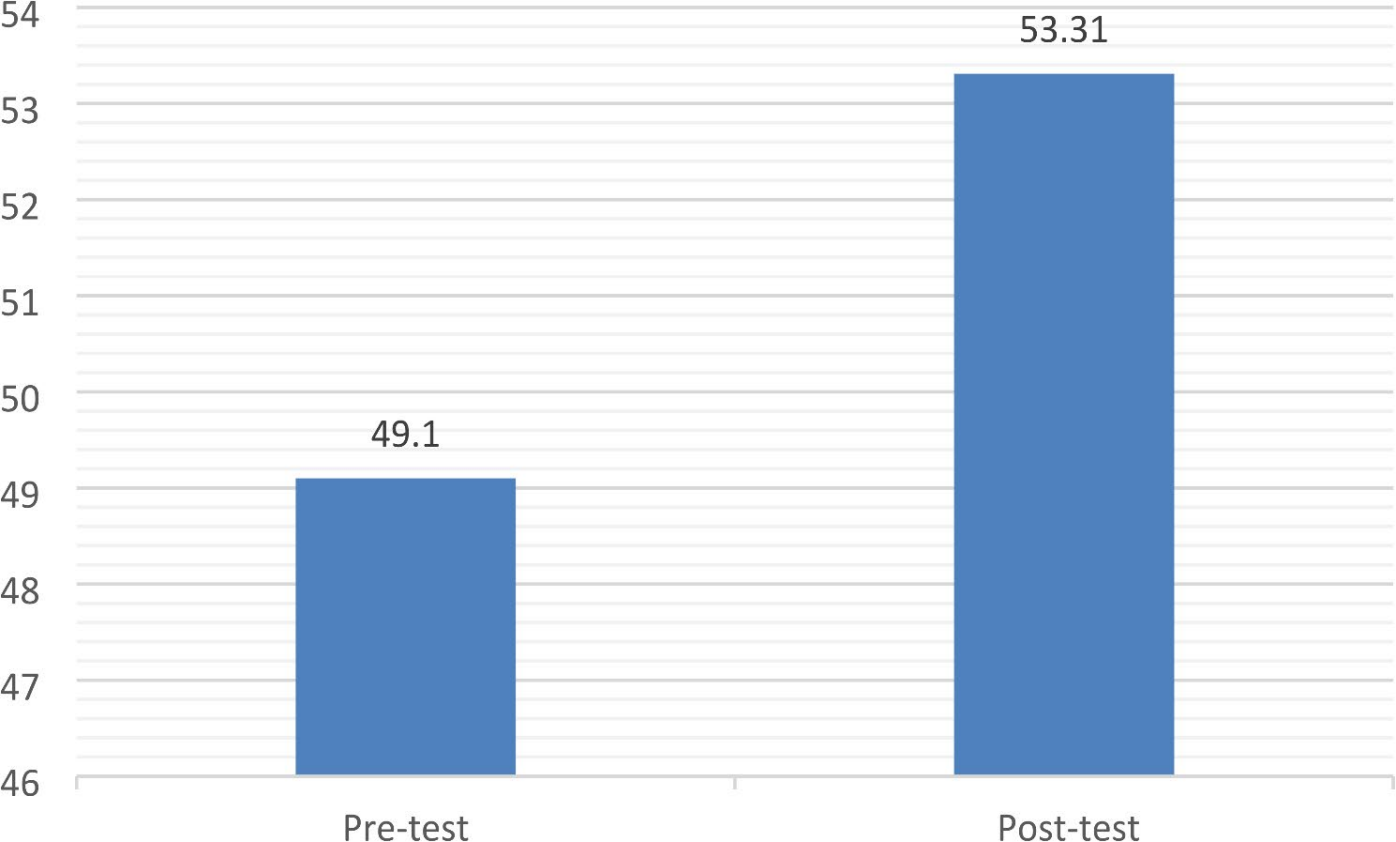
Mean pre-and post-test EBP-B scale scores (*n* = 81)

The mean post-test EBP-B scale score was significantly higher than the mean pre-test score (see Fig. 3.: t(80) = -5.248; 95% CI: -5.81, -2.61, *p* < 0.001).

**Fig. 4:**
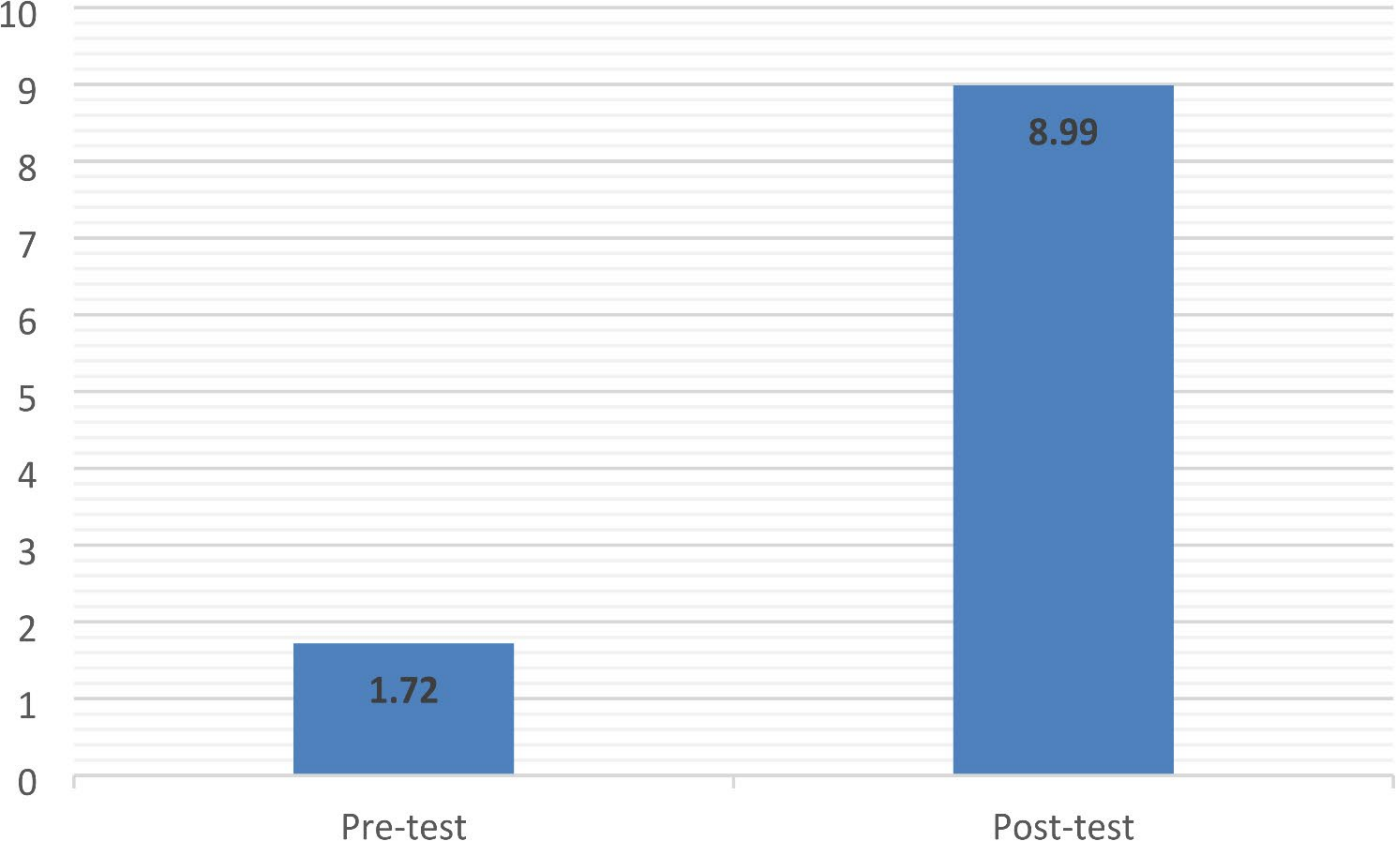
Mean pre- and post-test EBP-I scale scores (*n* = 81)

The mean post-test EBP-I scale score was significantly higher than the mean pre-test score (see Fig. 4.: t(80) = -6.133; 95% CI: -9.63 - -4.91, *p* < 0.001).

### Correlations between the EBP beliefs and implementation scores at baseline and the end of the study

Pearson correlations were computed to examine whether there were any statistically significant associations between baseline and end-of-the-study EBP-B scores and student nurses’ EBP-I scores. Indeed, the correlation was positive, meaning that higher EBP-B scores tended to have higher EBP-I scores. No significant correlation was found between EBP-B and EBP-I scores at baseline (*p* = 0.320). However, EBP-B and EBP-I scores showed a significant positive correlation at the end of the study (*p* < 0.001) (Table 6), indicating that higher EBP-B scores were associated with higher EBP-I scores.

**Table 6:** Intercorrelations between total EBP-B and EBP-I scores at baseline and the end of the study (*n* = 81)

	Pearson correlation	95% CI		*p*-value
		Lower B	Upper B	
EBP-B score / EBP-I score at baseline	0.112	-0.110	0.322	0.320
EBP-B score / EBP-I score at the end of the study	0.521	0.339	0.662	< 0.001

### Multivariate linear regressions of total EBP-B and EBP-I scores

Simultaneous multivariate two-step linear regression was conducted to investigate the best predictors of total EBP-B and EBP-I scores at the end of the study. The computed results were adjusted for the total EBP-B and EBP-I scores at baseline. Additionally, we computed how well EBP-B scores at baseline predicted the EPB-I total scores at baseline and at the end of the study.

#### EBP-Beliefs total scores

A two-step linear regression was computed to investigate how well the combination of clinical internships (acute care and long-term care settings) and the theory-based component on EBP significantly predicted EBP-B scores at the end of the study, adjusted for the EBP-B scores at baseline. The internship in community care was excluded because of the small sample among the respondents (*n* < 10) [48]. The combination of clinical internships and the theoretical-based component did not significantly predict EBP-B scores at the end of the study, with an R^2^ = 0.057 (*F* (3/77) = 1.540; *p* = 0.211) and an adjusted R^2^ of 0.020. When the EBP-B scores at baseline were added, they significantly improved the prediction of EBP-B scores at the study’s end, with an R^2^ change of 0.349 (*F* (1, 76) = 34.192; *p* < 0.001) and with an adjusted R^2^ of 0.351. According to Cohen, this is a moderate effect [53, 54]. The beta weights and significance values, presented in Table 7, indicate which variables contribute most to predicting the EBP-B scores at the end of the study. The combination of clinical internships, acute care and long-term care settings, and the theoretical-based component significantly predicted EBP-B scores at baseline when they were entered together as one predictor (t = 2.317 (df = 1/77); *p* = 0.023; 95% CI: 0.495, 6.561).

**Table 7:** Multivariate linear regression analysis of clinical internships (acute care and long-term care settings) and the educational programme’s theory-based component and the total EBP-B scores at the end of the study, adjusted for the EBP-B scores baseline (*n* = 81)

Variables	B	Std. Error	Exp(B)	t	Sig.	95% confidence interval	
						LB	UB
**STEP 1**							
Intercept	49.762	2.859		17.407	< 0.001	44.069	55.454
Theory-based component	3.302	1.821	0.206**	1.813	0.074	-0.324	6.929
Most internships in acute care	2.718	3.045	0.172	0.893	0.375	-3.345	8.781
Most internships in long-term care	2.091	3.108	0.130	0.673	0.503	-4.098	8.280
**STEP 2**							
Intercept	18.007	5.933		3.035	0.003	6.190	29.454
Theory-based component	3.528	1.523	0.220**	2.317	0.023*	0.495	6.561
Most internships in acute care	3.382	2.548	0.215	1.327	0.188	-1.693	8.456
Most internships in long-term care	2.271	2.598	0.141	0.874	0.385	-2.904	7.446
EBP Beliefs score at study entry	0.637	0.109	0.542	5.847	< 0.001	0.420	0.853

Note. * significant *p* < 0.05; ** contributing to predicting EBP-B scores at the end of the study

#### EBP-Implementation total scores

The linear regression to investigate whether the combination of clinical internships (acute care and long-term care settings) and the theory-based component on EBP could predict EBP-I scores at the end of the study, adjusting for the EBP-I scores at baseline, found that they did so significantly, with an R^2^ = 0.223 (*F* (3/77) = 7.384; *p* < 0.001) and an adjusted R^2^ of 0.193. The internship in community care was excluded because of its small sample of respondents (*n* < 10) [48]. Additionally, the theory-based component also significantly predicted nursing students’ EBP-I scores at the end of the study (*p* < 0.001, 95% CI: 5.136, 14.162) (Table 8). When the EBP-I scores at baseline were added, they slightly improved the prediction of EBP-I scores at the end of the study, with an R^2^ change of 0.270 (*F* (1, 76) = 4.854; *p* = 0.031) and an adjusted R^2^ of 0.232. According to Cohen, this is a moderate effect [48, 49]. The beta weights and significance values, presented in Table 8, indicated which variables contributed most to predicting EBP-I scale scores at the end of the study. This was when clinical internships (acute care and long-term care settings) and the theory-based component on EBP were entered together as one predictor.

**Table 8:** Multivariate linear regression analysis of internships, the educational programme’s theory-based component and the EBP-I scores at the end of the study, adjusted for EBP-I scores at baseline (*n* = 81)

Variables	B	Std. Error	Exp(B)	t	Sig.	95% confidence interval	
						LB	UB
**STEP 1**							
Intercept	-0.368	3.558		-0.104	0.918	-7.453	6.716
Theory-based component	9.649	2.266	0.439**	4.257	< 0.001*	5.136	14.162
Most internships in acute care	6.036	3.790	0.279	1.593	0.115	-1.510	13.582
Most internships in long-term care	5.998	3.868	0.271	1.551	0.125	-1.704	13.701
**STEP 2**							
Intercept	-2.330	3.584		-0.650	0.518	-9.469	4.808
Theory-based component	9.472	2.213	0.431**	4.280	< 0.001*	5.064	13.880
Most internships in acute care	6.815	3.715	0.315	1.835	0.070	-0.584	14.214
Most internships in long-term care	7.084	3.807	0.320	1.861	0.067	-0.498	14.666
EBP Implementation scores at baseline	0.705	0.320	0.218	2.203	0.031	0.068	1.343

Note. * significant *p* < 0.05; ** contributing to predicting EBP-I scores at the end of the study

### Total EBP-I scores were predicted by total EBP-B scores at the end of the study

A linear regression (Table 9) found that total EBP-B scores at the end of the study significantly predicted EBP-I scores at the end of the study when adjusted for baseline total EBP-I scores, with an R^2^ = 0.272 (*F* (1/79) = 29.447; *p* < 0.001) and an adjusted R^2^ of 0.262. When baseline EBP-I scores were added, they slightly improved the prediction of EBP-I scores at the end of the study, with an R^2^ change of 0.297 (*F* (1, 78) = 2.872; *p* = 0.097) and with an adjusted R^2^ change of 0.279. According to Cohen, this is a moderate effect [48, 49].

**Table 9:** Multivariate linear regression analysis of EBP-I scores predicted by the total EBP-B scores at the end of the study, adjusted for EBP-I scores at baseline (*n* = 81)

Variables	B	Std. Error	Exp(B)	t	Sig.	95% confidence interval	
						LB	UB
**STEP 1**							
Intercept	-29.117	7.098		-4.102	< 0.001	-43.245	-14.988
Total EBP-B scores at the end of the study	0.715	0.132	0.521**	5.427	< 0.001*	0.453	0.977
**STEP 2**							
Intercept	-28.744	7.019		-4.095	< 0.001	-42.718	-14.771
Total EBP-B scores at the end of the study	0.691	0.131	0.504**	5.277	< 0.001*	0.430	0.952
EBP-I scores at study entry	0.523	0.309	0.162	1.695	0.094	-0.091	1.138

Note. * significant *p* < 0.05; ** contributing to predicting EBP-I scores at the end of the study

## Discussion

The present study investigated the beliefs about EBP and the frequency of its implementation among nurses who had completed their BSN at the School of Health Sciences of Valais/ Wallis in Switzerland. We measured the hoped-for progression in their beliefs about EBP and the frequency of its implementation using the EBP-B and EBP-I scales, administered before and after their multi-component education programme on EBP. This program included their standard curriculum on research methodology and EBP, the usual clinical internships and a new workshop-based EBP intervention. We found significant increases in total EBP-B scale scores (an average of more than 4 points better, plus higher mean scores for 14 of the 16 items) and in total EBP-I scale scores (an average of more than 6 points better and, not surprisingly, higher mean scores for all 17 items). The most significant contribution to these positive changes in scores was from the theory-based component of the student nurses’ education, indicating a strong association between our multi-component EBP educational programme and increased frequency of EBP implementation at the end of the study. Indeed, these findings were in line with those of Kim et al.. among nursing students in South Korea who participated in a multi-component EBP teaching programme including clinical internships. Their EBP intervention demonstrated positive effects on formulating clinical questions, searching the relevant literature and critical appraisal skills, and their intervention group had statistically significant higher post-test EBP skills scores (*p* < 0.001) than the control group [29]. Our results also corroborated Oh and Yang (2019), who reported significantly better EBP skills in an EBP educational intervention group of 21 undergraduate nursing students compared to a control group of 24 students, including significant effects in EBP knowledge, self-efficacy, resource utilisation and database utilisation (*p* < 0.01) [50]. Similar beneficial results were illustrated in a pilot study by Reid et al.. using a quantitative pre–post-test design and demonstrating that using an educational intervention positively impacted EBP beliefs and the frequency of EBP implementation, as measured using the EPB-B and EBP-I scales [51].

Our findings revealed that our multi-component educational programme on EBP helped the participating undergraduate students to integrate scientific knowledge and apply EBP in practice. At the beginning of the study, EBP-B scores reflected students’ positive attitudes toward the concept, but EBP-I scale scores were lower, surely because they had not had opportunities to implement it on wards. Our findings indicated that undergraduate BSN students predominantly developed their beliefs about EBP and its frequency of implementation through the theory-based component of the educational programme on EBP and less so from their clinical internships. The lack of opportunities to implement EBP in clinical practice before beginning their studies naturally negatively influenced the student nurses’ baseline EBP-I scores, as previously highlighted by Key et al.. and Larsen et al. [18, 52]. Although we were surprised by our finding that the theory-based component of the educational programme on EBP had more impact on their EBP scores than did clinical internships, other authors have underlined that EBP should be taught in all healthcare curricula and then applied during clinical internships [53, 54, 55]. The integration of EBP into regional healthcare centres still seems to be in an embryonic phase in Switzerland, as reported by Pereira et al.., Perruchoud et al.., and Verloo et al. [43, 56, 57].

The educational programme in EBP covered the entire EBP process, requiring the development of skills in formulating clinical questions, finding sources of evidence, critically appraising that evidence and applying findings to clinical practice. BSN students are continuously exposed to EBP processes and content during their theory-based education. The transfer of knowledge about EBP should be completed through their clinical internships. Indeed, nursing students should be encouraged and mentored to use EBP in their practice-based education, not just to consider it an academic exercise.

Undergraduate nursing students will be required to adhere to best practice and competencies throughout their professional careers. Coomarasamy and Khan’s systematic review of 23 studies found that stand-alone classes on the theory of EBP or courses on critical appraisal skills improved knowledge but that integrated clinical internships enhanced students’ skills, attitudes and behaviours [58]. The use of EBP was integrated into all our students’ various clinical internships, and they resulted in different outcomes. Our findings and the literature suggest that EBP should be integrated into every BSN course as a cross-cutting element [59, 60, 61, 62]. Integrating the concepts and processes of EBP into every clinical internship, throughout their curriculum, will be more likely to produce nurses who can deliver evidence-based care, as reported by O’Toole [63] and by Key et al. [18]. We believe that the approach used by the School of Health Sciences of Valais/ Wallis promotes research as a fundamental educational objective and will influence students’ abilities to base their care on EBP. An educational setting’s attitudes and organisation can either help or hinder the development of a culture that supports moves toward EBP [29, 64].

Previous publications have reported on the difficulties of integrating content on EBP into severely crowded BSN curricula [29, 65, 66]. There is probably a need to revise these, fully integrating courses on the theory of EBP and applying its processes during clinical internships. Any curriculum revisions should encourage students to learn the essential knowledge and skills associated with using EBP and to develop their confidence in incorporating it into their daily work. Linking theory to clinical activities where students were expected to demonstrate EBP in real-world healthcare settings should not require major curriculum changes. Brown et al.. identified how courses integrating EBP clinically and including strong partnerships between academic and clinical institutions, could enhance students’ confidence in using EBP immediately and into the future [34]. Our findings aligned with the consensus that reinforcing training in the theory and practice of EBP should occur early on in BSN programmes so that students have opportunities to implement it throughout their curricula and at the bedside [21]. Nevertheless, improving undergraduate nurses’ beliefs in EBP and their skills to implement it could be a key strategy to support the concept and achieve improved clinician, patient and organisational outcomes. Several studies have reported that nurses today should be sufficiently prepared to embrace EBP in whatever their healthcare setting, and they identified educational preparation as a significant contributing factor [29]. Introducing EBP as a core nursing concept creates a framework to guide students’ practice and prepare them for future care-related challenges. Graduate nurses are challenged by today’s complex and ever-changing healthcare environments, and grounding them in how to use research and embedding EBP programmes into their curricula could ensure that future leaders of the profession contribute toward safe, evidence-based healthcare [67]. Our findings support the idea that building a culture of EBP in educational settings could lead to nurses playing key roles in integrating the concept into their organisational cultures [68].

### Strengths and limitations

This study’s main strength was that there has been little published research on educational programmes and interventions regarding EBP and their associations with undergraduate nursing students’ clinical internships [69]. We have presented new evidence on the relevance of developing skills in EBP among future nurses.

Our investigation also had some limitations. The EBP-B and EBP-I scales are self-reporting instruments; thus, a lack of objectivity may have led students to under- or overestimate scores and may not have reflected their actual abilities to implement EBP at the bedside [22]. However, various authors have reported strong correlations between self-reported and objective assessments of EBP knowledge and competencies [70, 71].

Additionally, the small sample size (*n* = 81) and the single study site limit the generalizability of our findings. While our results provide valuable insights, they should be interpreted with caution, as they may not fully represent the diversity of nursing students’ experiences in other settings. Future research should involve larger samples across multiple sites to strengthen the robustness and external validity of the findings.

The choice of the EBP-B and EBP-I scales designed for nurses, rather than the student-specific versions, represents another limitation. Although these scales have been validated and culturally adapted in French and German, their content was originally developed for practicing nurses, which may influence how nursing students perceive and report their beliefs and practices related to EBP. The absence of validated student-specific versions in the study languages justified this choice, but certain wording or concepts may not fully reflect the competency level of students in training, potentially introducing bias and limiting comparability with studies using student-adapted versions. Future research should focus on translating and validating these student versions to improve measurement accuracy for this specific population.

### Implications for education

Preparing the nurses of the future, nurses who believe in and are skilled at implementing EBP, is paramount to ensuring safe, effective, high-quality patient care outcomes. To achieve this, undergraduate curricula in nursing should integrate the theory and processes of EBP across every module, and the concept should be promoted during students’ clinical internships. Raising expectations about what students should know and the skills they should have at various points in their education, and developing assignments that reflect those expectations, should be part of the innovative teaching strategies that facilitate student engagement with EBP. Based on our findings and those of similar studies, we suggest that EBP should be embedded into student nurses’ multi-component educational programmes and promoted during their clinical practice internships as part of their undergraduate curricula.

## Conclusion

In line with the goals and vision of both Switzerland’s Federal Law on the Healthcare Professions (LPsan) and its healthcare system, it is essential that nursing curricula across the country—especially when they are taught in three different national languages—embed the teaching of evidence-based practice (EBP) into every theory-based classroom module and into every practical clinical experience. Our findings add to a slowly growing body of work reporting on how multi-component educational programmes on EBP are to be recommended for improving future nurses’ beliefs in the concept and their frequency of implementation. Indeed, it is expected that this could have a significant impact on nurses’ day-to-day practice and, ultimately, the quality of healthcare and outcomes for patients. Combining the theoretical teaching of EBP with its practical application during clinical internships contributed to improving students’ EBP-Beliefs and EBP-Implementation scale scores; however, it did not allow us to draw any conclusions about EBP’s implementation at the patient’s bedside.

This investigation highlighted the need to promote education in EBP in Bachelor of Sciences in Nursing curricula. Fostering a culture that embraces EBP will prepare a future workforce that can deliver evidence-based care, and this will be mandatory for improving nursing standards in all higher education institutions.

## Electronic supplementary material

Below is the link to the electronic supplementary material.

Supplementary Material 1

Supplementary Material 2

## Data Availability

The datasets used and/or analysed during the current study are available from the corresponding author on reasonable request.

## Abbreviations

BSN: Bachelor of Sciences in Nursing
EBP: Evidence-based practice
EBP-B: Evidence-Based Practices Belief scale
EBP-I: Evidence-Based Practices Implementation scale
ECTS: European Credit Transfer and Accumulation Scheme
HES-SO: University of Applied Sciences and Arts Western Switzerland
IB: Internal Board ethical committee
PEOT: Population, patient or problem; exposure; outcomes or themes; time
PICOT: Population or patient problem; intervention; comparison; outcome; time
