# Supplementary material for: Enhancing beliefs and implementation of evidence-based practice among undergraduate nurses using a multi-component educational programme: a pre–post study
Source: BMC Med Educ. 2025 Apr 14;25:531. doi: 10.1186/s12909-025-07121-x (PMC11998436; doi:10.1186/s12909-025-07121-x)
Supplement: Supplementary file 2 — Supplementary Material 2 [file 12909_2025_7121_MOESM2_ESM.docx]

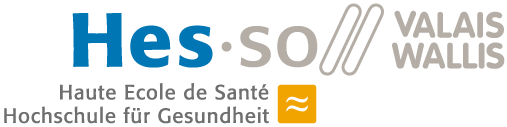


[The original French version was translated in English for publication purposes.]

Sion, 29 September 2017

Dear Sir/Madam,

Dear student,

The School of Health Sciences of HES-SO Valais/Wallis would like to conduct a research among students entitled “Enhancing beliefs about and implementation skills in evidence-based practice among undergraduate nurses using a multi-component educational programme : a pre-post study”.

We are interested in your opinion. We are asking for your participation in order to identify your beliefs and implementation skills in evidence-based practice. This is a pre-post study, and data will be collected at both the beginning and the end of your curricula to assess changes over time.

We are providing you with two questionnaires accompanied by a brief definition, which should take no more than 20 minutes to complete. The data will be treated in the strictest confidence and in the interests of this single survey. Participation is entirely voluntary.

We know that your time is precious, and we would like to thank you in advance for your involvement, which will help us to better understand your training path.

Please do not hesitate to contact us if you have any further questions.

Best regards,

Filipa Pereira

Brigitte Lehmann-Wellig

Henk Verloo


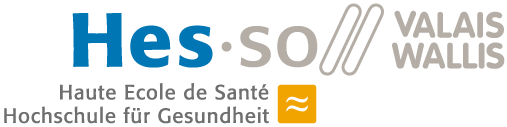


EBP Questionnaire

Before completing this questionnaire, please read the following carefully. We are interested in the concept of evidence-based practice (EBP), which is also known as research-based practice, best practice based on evidence, evidence-informed practice, practice based on scientific evidence, evidence-based medicine (EBM) or evidence-based nursing (EBN).

Evidence-based practice includes the following components:

- External Evidence from Research, Opinion Leaders and Expert Panels;

- Clinical Expertise;

- Patient Preferences and Values.

By participating in this study, you certify that you have been duly informed of the aim of the research entitled “Enhancing beliefs about and implementation skills in evidence-based practice among undergraduate nurses using a multi-component educational programme : a pre-post study” and that, if needed, you have been able to pose questions to the research team. You agree to the publication of this research's results in an anonymous manner, guaranteeing that your identity remains untraceable. You have the option not to participate.

Thank you for your valuable participation.

Please answer all the following items by ticking the box that best corresponds to your situation. The data will be processed anonymously.

Registration number : ……………………………………………..

Sex : _1_ Male _2_ Female

Age : ……………………….. years old

Registration site : _1_ Sion _2_ Visp

Training year : _1_ 1^st^ year _2_ 2^nd^ year _3_ 3^rd^ year

If remediations, how many years have you been on the programme? ..........................years

Clinical internships completed (several answers possible):

_1_ Acute care (hospital care)

_2_ Long-term care (rehabilitation, nursing homes, long-term healthcare facilities)

_3_ Community healthcare (outpatient primary care and community healthcare settings)

Have you ever heard of evidence-based practice?

_1_ No

_2_ Yes

Where have you heard about evidence-based practice (several answers possible)?

_1_ During basic training in nursing

_2_ During clinical internships

_3_ Personal context (conferences, newspapers, reading articles, etc.)

_4_ As part of my professional activity alongside my studies

_5_  Others : ………………………………………………………………………………………..

Please answer all the questions on the following pages. We thank you for your valuable collaboration.


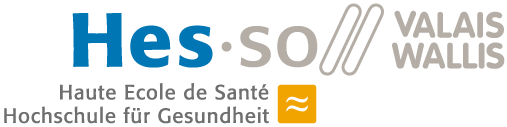


**EBP SCALES**

[Melnyk et al.’s self-reported EBP Beliefs and Implementation Scales were used (1). The scales were completed in French (2) or German (3) at the two school sites located in the canton of Valais, corresponding to the French- and German-speaking regions, respectively.]

1. Melnyk BM, Fineout-Overholt E, Mays MZ. The evidence-based practice beliefs and implementation scales: psychometric properties of two new instruments. Worldviews Evid Based Nurs. 2008;5(4):208-16.
2. Verloo H, Desmedt M, Morin D. Adaptation and validation of the Evidence-Based Practice Belief and Implementation scales for French-speaking Swiss nurses and allied healthcare providers. J Clin Nurs. 2017;26(17-18):2735-43.
3. Kerwien-Jacquier E, Verloo H, Pereira F, Peter KA. Adaptation and validation of the evidence-based practice beliefs and implementation scales into German. Nurs Open. 2020;7(6):1997-2008.
